# Uterine Rupture on Invasive Molar Pregnancy Revealing a Choriocarcinoma: Case Report from a Low‐Income Country

**DOI:** 10.1155/crog/6055745

**Published:** 2026-02-27

**Authors:** Rakotomalala Nivoarimelina Zoly, Refeno Valéry, Ramarokoto Malalafinaritra Patrick Marco, Randaoharison Pierana Gabriel, Rafaramino Florine

**Affiliations:** ^1^ Mother-Child Complex, Professor Zafisaona Gabriel University Hospital, Mahajanga, Boeny Region, Madagascar; ^2^ Oncology Department, Professor Zafisaona Gabriel University Hospital, Mahajanga, Boeny Region, Madagascar; ^3^ Faculty of Medicine, University of Mahajanga, Mahajanga, Boeny Region, Madagascar; ^4^ Faculty of Medicine, University of Antananarivo, Antananarivo, Analamanga Region, Madagascar, univ-antananarivo.mg

**Keywords:** case report, choriocarcinoma, invasive mole, madagascar, uterine rupture

## Abstract

**Introduction:**

Gestational trophoblastic diseases are rare entities encompassing a wide spectrum of benign and malignant placental pathologies. Uterine rupture is primarily related to a uterine scar and, exceptionally, following an invasive mole or gestational choriocarcinoma. Our objective is to report a rare case of uterine rupture following an invasive mole revealing gestational choriocarcinoma.

**Case Presentation:**

A 23‐year‐old pregnant woman was admitted for genital bleeding complicated by hypovolemic shock. She presented with symptoms suggestive of a ruptured ectopic pregnancy. However, her hCG levels were markedly elevated, raising suspicion of gestational trophoblastic disease. A subtotal hysterectomy with bilateral salpingo‐oophorectomy and bladder repair was performed. Due to the lack of histopathology, the diagnosis of choriocarcinoma was clinically and biochemically inferred. Exclusive chemotherapy using the methotrexate–folinic acid protocol was initiated. The patient was lost to follow‐up after the first course.

**Conclusion:**

The reported case is generally consistent with data from the literature. The practitioner must emphasize the highly curable nature of the disease to optimize patient adherence until the end of the treatment plan. Early diagnosis must be optimized to allow for conservative treatment.

**Summary**

• Uterine rupture can rarely reveal gestational choriocarcinoma.
• High hCG levels in first‐trimester bleeding should prompt suspicion.
• Early recognition and adherence to chemotherapy can achieve cure even in low‐resource settings.

## 1. Introduction

Gestational trophoblastic diseases are rare entities encompassing a wide spectrum of benign and malignant pathologies of the placenta. Molar pregnancy is an anarchic development of pseudocystic edematous chorionic villi. It may be avascular without a fetus (complete mole) or with an abnormal fetus in the process of lysis (partial mole). It is said to be invasive when it invades the myometrium and/or the broad ligament by villous structures with hyperplasia of cytotrophoblastic and syncytial elements [1, 2]. Choriocarcinoma is a malignant tumor of epithelial origin at the expense of trophoblastic tissue. It is rare, representing less than 1% of cancers of the female genital tract. Nearly half of choriocarcinomas develop after a molar pregnancy. This cancer is highly sensitive to anticancer chemotherapy, allowing to obtain a complete remission without requiring surgery and to maintain the capacity for subsequent pregnancies [3, 4]. Usually, the circumstances of discovery are related to locoregional invasion and metastatic extension, allowing time to implement medical treatment. In the literature, there have been reported about 10 cases of choriocarcinoma revealed by a uterine rupture [5–7]. We report the case of a choriocarcinoma revealed by a uterine rupture in order to share the diagnostic, therapeutic, and prognostic challenges of this pathology in low‐income countries.

## 2. Case Presentation

This was a 23‐year‐old patient admitted to the emergency department of the Mother‐Child Complex at the Professor Zafisaona Gabriel Hospital in Mahajanga in early November for genital bleeding complicated by hypovolemic shock. She was the mother of a child born naturally 3 years earlier. The date of her last menstrual period was unknown. She mentioned a notion of spontaneous miscarriages occurring in early October (1 month earlier) with bleeding and clots. She had not consulted a health center. Since then, she reportedly experienced blackish bleeding and minimal pelvic pain that was becoming increasingly intense. On the day of admission, she presented a significant deterioration in her general condition, prompting her arrival at the emergency room. On admission, the woman was agitated due to pain and presented with hypovolemic shock and generalized pallor. She was not feverish. The abdomen was distended and rigid. It was impossible to assess the uterus. Speculum examination revealed a purplish cervix with slight bleeding from the endocervix. Vaginal examination found a slightly enlarged uterus and sharp, localized pain upon deep palpation of the rectouterine pouch (Douglas’ sign). The urine pregnancy test was positive. Pelvic ultrasound revealed a hemoperitoneum, with a floating uterus containing blood, two ovaries with anechoic septate cysts and a poorly defined supravesical lesion. The ultrasound did not reveal any abdominal lymphadenopathy or visceral lesions suggestive of metastases. The patient presented with severe anemia at 5 g/dL of hemoglobin. The preoperative hCG level was 543,000 mIU/L.

**Figure 1:**
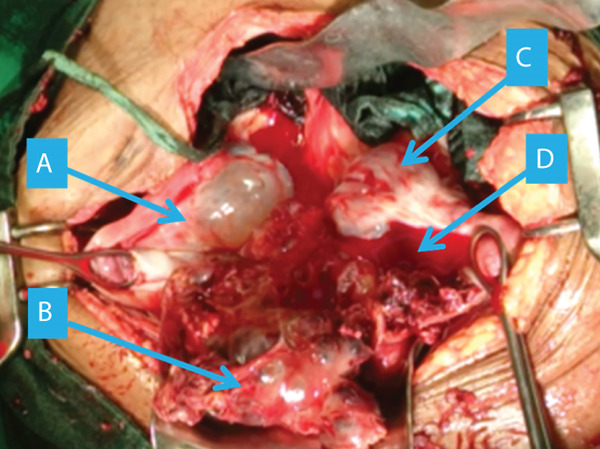
Overview at opening ((a) right ovary, (b) molar tissue, (c) uterus, and (d) hemoperitoneum).

**Figure 3:**
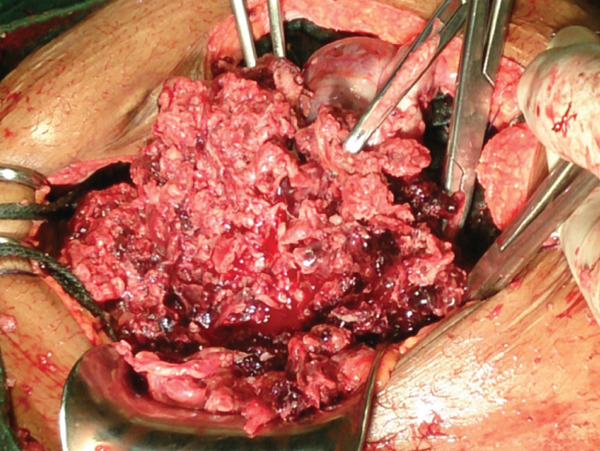
Molar tissue with vesicles.

The clinical picture initially suggested a ruptured ectopic pregnancy. We performed an emergency laparotomy, which revealed a 3000‐mL hemoperitoneum (Figure 1) with vesicoparietal adhesions elevating the bladder and two polycystic ovaries with citrin‐like fluid content (Figure 2). The uterus was torn at the antero‐isthmic level through which tissue mixed with vesicles protruded to the bladder dome (Figure 3). A subtotal hysterectomy with bilateral salpingo‐oophorectomy and bladder repair was performed. The hysterectomy was subtotal because the cervix was macroscopically healthy, and the operative time was shortened due to the hypovolemic shock state. Bilateral salpingo‐oophorectomy was performed in the young woman because the two ovaries were polycystic and suspicious intraoperatively. Two bags of packed red blood cells and one bag of fresh frozen plasma were transfused during the procedure. There were no intraoperative or immediate postoperative complications.

Figure 2Two polycystic ovaries ((a) right ovary and (b) left ovary).

**Figure:**
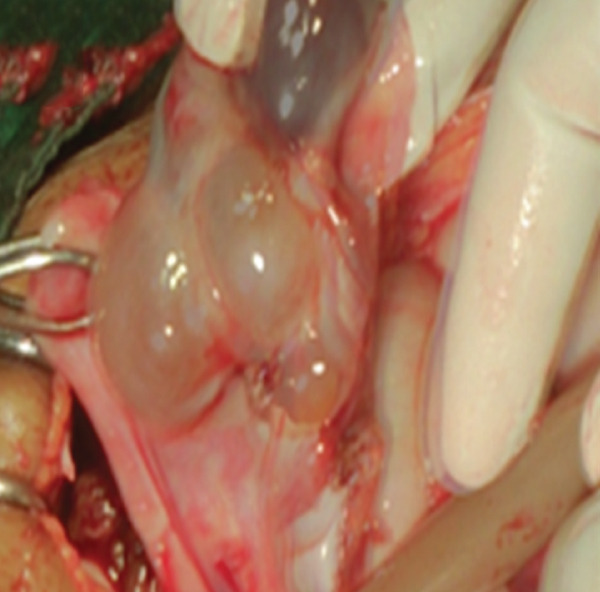
(a)

**Figure:**
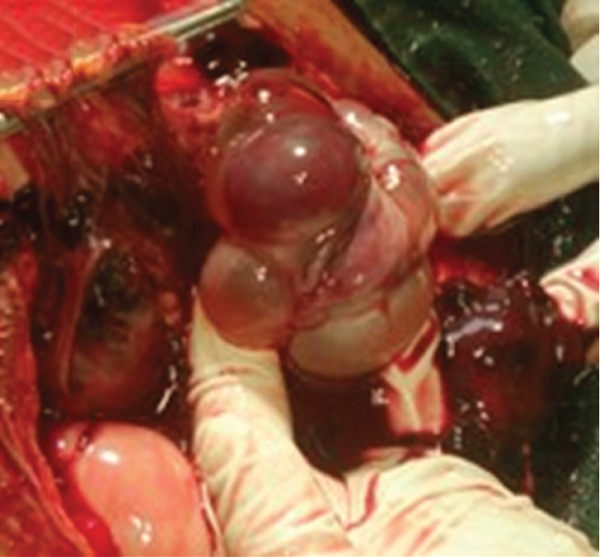
(b)

Histopathology of the surgical specimen was not performed because it would have required transporting the surgical specimens to the capital at the patient’s expense, for which financial means were lacking. The postoperative period was marked by persistent vaginal bleeding. There was an initial decrease in hCG levels in the first week after surgery (4800 mIU/mL) followed by an increase from the third week. hCG level reached 418,000 mIU/mL 1 month and 1 week postoperatively. Due to the lack of histopathology, the diagnosis of choriocarcinoma was clinically and biochemically inferred. The thoraco‐abdomino‐pelvic CT scan could not be performed. Aternatively, a chest X‐ray was performed and did not reveal any pleuropulmonary lesions suggestive of distant metastatis. The International Federation of Gynecology and Obstetrics (FIGO) prognostic score was 6. The disease was classified as “low risk.” Exclusive anticancer chemotherapy was proposed according to the methotrexate protocol: methotrexate 1 mg/kg on Days 1, 3, 5, and 7 in alternation with folinic acid 0.1 mg/kg on Days 2, 4, 6, and 8. The chemotherapy is planned to be repeated every 2 weeks until the hCG level was negative and then add two additional courses. The patient was lost to follow‐up after the first course of chemotherapy.

## 3. Discussion

### 3.1. Epidemiology

During an invasive mole, the molar tissue crosses the thickness of the myometrium and leads to uterine perforation and massive intra‐abdominal hemorrhage. Uterine rupture complicating a molar pregnancy is rare [8]. Our case was the first case encountered in our department in the 15 years since its opening (one case out of 42,540 pregnancies admitted). Most cases of uterine rupture on invasive mole reported in the literature had presented with hypovolemic shock in a context of uterine fragility in multiparous women aged around 30. The cases described in the literature had benefited from curettage following the suspicion of a hydatidiform mole on ultrasound [8–10]. Our patient was a young mother of 23 years who had given birth only once and without prior cesarean.

### 3.2. Diagnostic Challenge

The clinical picture as well as the result of the emergency ultrasound were misleading and suggested a ruptured ectopic pregnancy. Differential diagnosis includes gestational trophoblastic diseases, placenta accreta spectrum, and scar pregnancy [11, 12]. Other authors have reported invasive hydatidiform mole mimicking ectopic pregnancy. Xiao et al. reported a series of 14 cases of invasive hydatidiform mole mimicking ectopic pregnancy observed over 15 years. Their identical clinical, imaging, and biological signs make differentiation difficult. The definitive diagnosis is made by histopathology. Nevertheless, the diagnosis of gestational trophoblastic tumors should be prioritized in cases of elevated hCG without a confirmed normal pregnancy and with the presence of metastases in a woman who has been pregnant [13–15].

The diagnosis was only made after the detection of tissue containing a few vesicles crossing the uterus and invading the bladder associated with polycystic ovaries and a very high hCG level. Indeed, the existence of abnormal myometrial invasion is often difficult to assess on pelvic ultrasound [8, 9]. Molar pregnancy can manifest clinically by first‐trimester vaginal bleeding with or without evacuation of vesicles associated with an exaggeration of sympathetic signs of pregnancy and a uterus larger than the gestational age. It is suspected in the presence of ultrasound images of “honeycomb” vesicles and inconstant bilateral ovarian cysts with an hCG level exceeding 100,000 IU/L. Today, ultrasound and serum hCG dosage often allow the diagnosis of molar pregnancies before surgery. The diagnostic performance of ultrasound is around 90% for complete moles and around 30% for partial moles. Serum hCG is elevated in almost 90% of complete moles, but in less than 10% of partial moles. We remind you that only the histopathology of curettage products can confirm the diagnosis of molar pregnancy and that it must be done systematically in order to optimize the subsequent management [2].

### 3.3. Specific Treatment and Follow‐Up

The specific management of choriocarcinoma discovered on uterine rupture varies according to the reported cases and according to the context. In the case reported by Belhaouz et al., in Morocco in 2024, the 43‐year‐old patient underwent a total hysterectomy, leaving a vaginal tumor infiltration. The choriocarcinoma was classified as low risk (FIGO score < 6), and a complete remission was obtained after chemotherapy according to the methotrexate‐folinic acid protocol [5]. In the case reported by Vinutha et al., in India in 2025, the 24‐year‐old patient underwent a salvage total hysterectomy. The FIGO score being equal to 7, she was referred to the oncology department for chemotherapy according to the protocol combining etoposide, methotrexate, actinomycin in weekly alternation with cyclophosphamide and vincristine (EMA‐CO) [6]. In the case reported by Gueye et al., in Senegal in 2016, the 25‐year‐old patient underwent an emergency laparotomy to see a tumor‐like uterine lesion infiltrating the intestinal loops. After dissection performed jointly with the visceral surgery team, a total hysterectomy was performed. Chemotherapy according to the EMA‐CO protocol was planned upon recovery from the operation but was not honored. The patient was readmitted 3 weeks later with a significant deterioration in her general condition. She died before being able to receive the chemotherapy [7]. In our case, the specific management of the patient was incomplete due to the limitations of the technical platform available and financial resources. Note that all the costs of care are at the charge of the patient and her family. After one course of chemotherapy, the patient was lost to follow‐up. According to the recommendations of the European Society of Medical Oncology, methotrexate monochemotherapy is the reference first‐line treatment for low‐risk gestational trophoblastic tumors. The recommended protocol is: methotrexate 1 mg/kg on Days 1, 3, 5, and 7 intramuscularly and calcium folinate (folinic acid) 0.1 mg/kg intramuscularly or 10 mg orally on Days 2, 4, 6, and 8. In this regimen, Day 1 recurs every 14 days. In case of contraindication or intolerance to methotrexate, actinomycin D monotherapy is recommended. The complete remission response rate is almost 100% in patients classified as low risk and 90% in patients classified as high risk. All stages combined, the risk of relapse is around 3% [3, 4]. Thus, the practitioner must emphasize the highly curable nature of the disease to optimize the patient’s adherence until the end of the therapeutic project.

### 3.4. Low‐Resource Considerations

Due to limited resources, key examinations such as thoraco‐abdomino‐pelvic CT scans, MRI, and histopathology could not be performed. The patient lacks social support and was unable to continue her cancer chemotherapy [4]. Malagasy national health insurance must be operationalized as soon as possible to allow access to care for the less fortunate.

### 3.5. Strength and Limit

The strength of this case report lies in its illustration of a rare, urgent, and serious clinical situation for which data in the literature remain limited. The study’s limitation is that the highly curable nature of choriocarcinoma could not be demonstrated because the patient was unable to receive the recommended treatment. The lack of histopathological confirmation is a critical diagnostic limitation and a challenge in the context of low‐resource healthcare systems. The unavailability of contrast‐enhanced CT scan may have influenced the FIGO prognostic scoring.

## 4. Conclusion

Uterine rupture complicating gestational choriocarcinoma constitutes a major diagnostic and therapeutic emergency. It is a rare situation whose signs can mimic those of a ruptured ectopic pregnancy. Emergency treatment for rupture is both surgical (hysterectomy) and medical (resuscitation). Curative treatment for choriocarcinoma involves exclusive anticancer chemotherapy. The prognosis is good when the treatment plan is properly implemented. We emphasize the importance of early detection of molar pregnancies to allow conservative management, the need for patient adherence, and the systemic support in low‐resource settings.

## Author Contributions

**Rakotomalala Nivoarimelina Zoly:** conceptualization (equal), investigation (equal), original draft preparation (equal), visualization (equal). **Refeno Valéry:** conceptualization (equal), investigation (equal), original draft preparation (equal), visualization (equal). **Ramarokoto Malalafinaritra Patrick Marco:** investigation (equal). **Randaoharison Pierana Gabriel:** supervision (equal), validation (equal), review and editing (equal). **Rafaramino Florine:** supervision (equal), validation (equal), review and editing (equal), project administration (lead).

## Funding

No funding was received for this manuscript.

## Consent

Written informed consent has been obtained and is available to the journal on request. The patient is anonymized according to the ICMJE guidelines.

## Conflicts of Interest

The authors declare no conflicts of interest.

## Data Availability

The data that support the findings of this study are available on request from the corresponding author. The data are not publicly available due to privacy or ethical restrictions.
